# Brain pathological changes during neurodegenerative diseases and their identification methods: How does QSM perform in detecting this process?

**DOI:** 10.1186/s13244-022-01207-6

**Published:** 2022-04-13

**Authors:** Farzaneh Nikparast, Zohreh Ganji, Mohammad Danesh Doust, Reyhane Faraji, Hoda Zare

**Affiliations:** 1 Medical Physics Research Center, Mashhad University of Medical Sciences, Mashhad, Iran; 2 Department of Medical Physics, Faculty of Medicine, Mashhad University of Medical Sciences, Mashhad, Iran

**Keywords:** Quantitative susceptibility mapping, Alzheimer’s disease, Iron, Magnetic susceptibility

## Abstract

The presence of iron is essential for many biological processes in the body. But sometimes, for various reasons, the amount of iron deposition in different areas of the brain increases, which leads to problems related to the nervous system. Quantitative susceptibility mapping (QSM) is one of the newest magnetic resonance imaging (MRI)-based methods for assessing iron accumulation in target areas. This Narrative Review article aims to evaluate the performance of QSM compared to other methods of assessing iron deposition in the clinical field. Based on the results, we introduced related basic definitions, some neurodegenerative diseases, methods of examining iron deposition in these diseases, and their advantages and disadvantages. This article states that the QSM method can be introduced as a new, reliable, and non-invasive technique for clinical evaluations.

**Graphical abstract:**

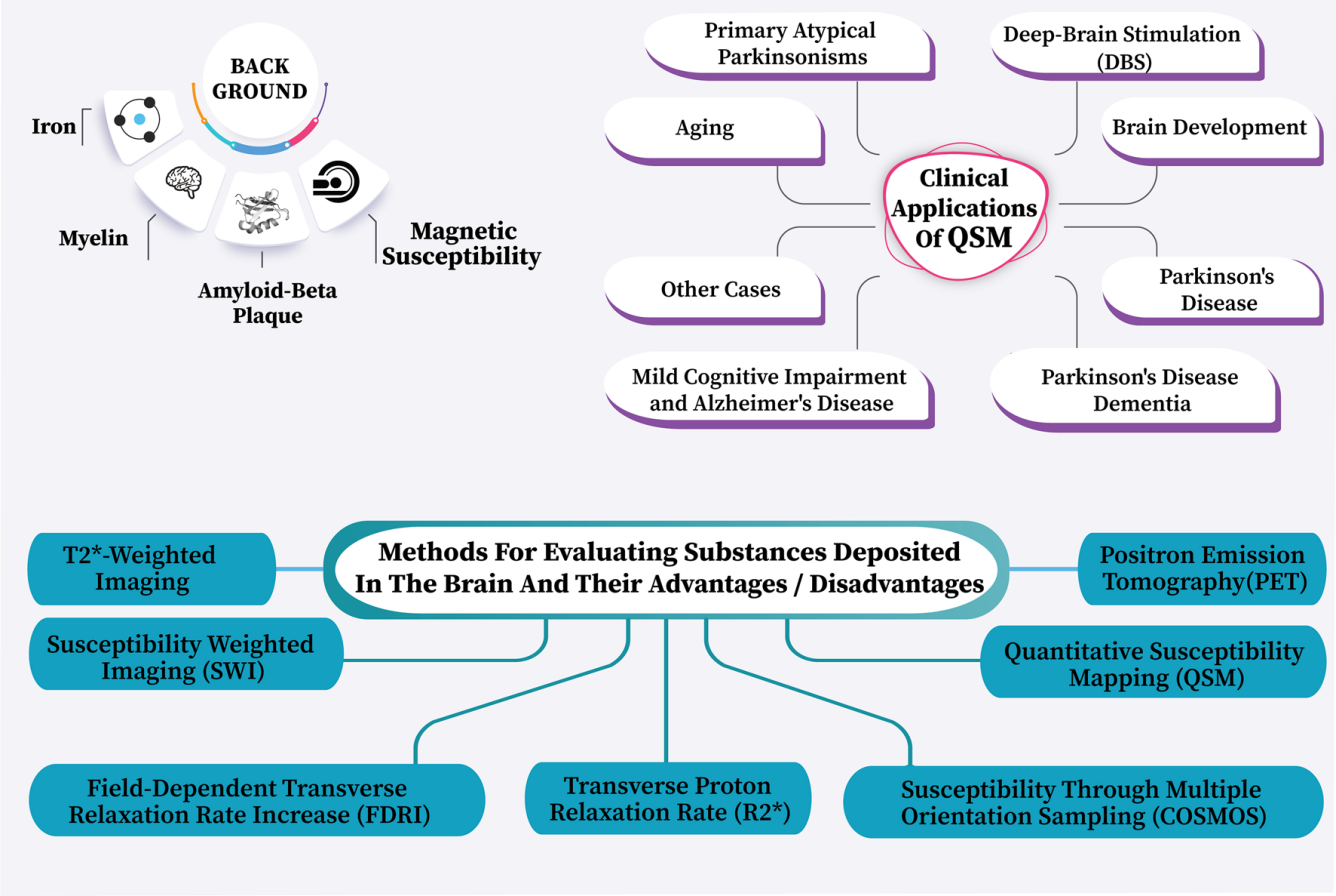

## Key points

- Identify the mechanism of the most significant pathological changes in the brain during neurodegenerative diseases.
- Diagnostic imaging methods to identify these pathological changes and the advantages and disadvantages of each.
- The clinical role of QSM and its function in the early detection of neurodegenerative diseases.

## Introduction

Iron plays a fundamental role in many biological processes such as cell growth, cell differentiation, protein expression, etc.

Despite the need for iron in the brain, its increase leads to toxic free radicals, oxidative stress, and nerve cell damage [1].

Iron deposition happens in various brain areas in some diseases of the nervous system and the aging process.

There are different imaging methods based on MRI for examining and measuring iron deposition in the brain, like T2*-weighted imaging (T2*WI), T2-weighted imaging(T2WI), relaxation rate (R2*), field-dependent relaxation rate increase (FDRI), and susceptibility-weighted imaging (SWI) [2].

Recently, techniques based on deep learning, machine vision, and medical image processing to diagnose neurodegenerative diseases have expanded; and today, quantitative susceptibility mapping (QSM) is one of the newest medical image post-processing techniques [3–8]. Iron Sediments cause changes in the magnetic susceptibility properties of brain tissue; QSM is a new and non-invasive method that works based on these changes to evaluate the values of iron accumulations.

It is a processing technique that can measure tissue susceptibility from various sequences such as Gradient echo sequences (GRE) and does not have many limitations [9].

This observational-descriptive article explains the main pathological factors in neurodegenerative diseases and how they work and move step by step to diagnostic imaging methods for these pathological factors and their advantages/disadvantages by summarizing the results of different articles.

Finally, a comparison between these methods and the QSM method was performed. This article aims to obtain sufficient information about the first brain changes in the early stages of cognitive disorders and their imaging methods and understand the QSM technique and its clinical applications.

## Materials and methods

**Fig. 1:**
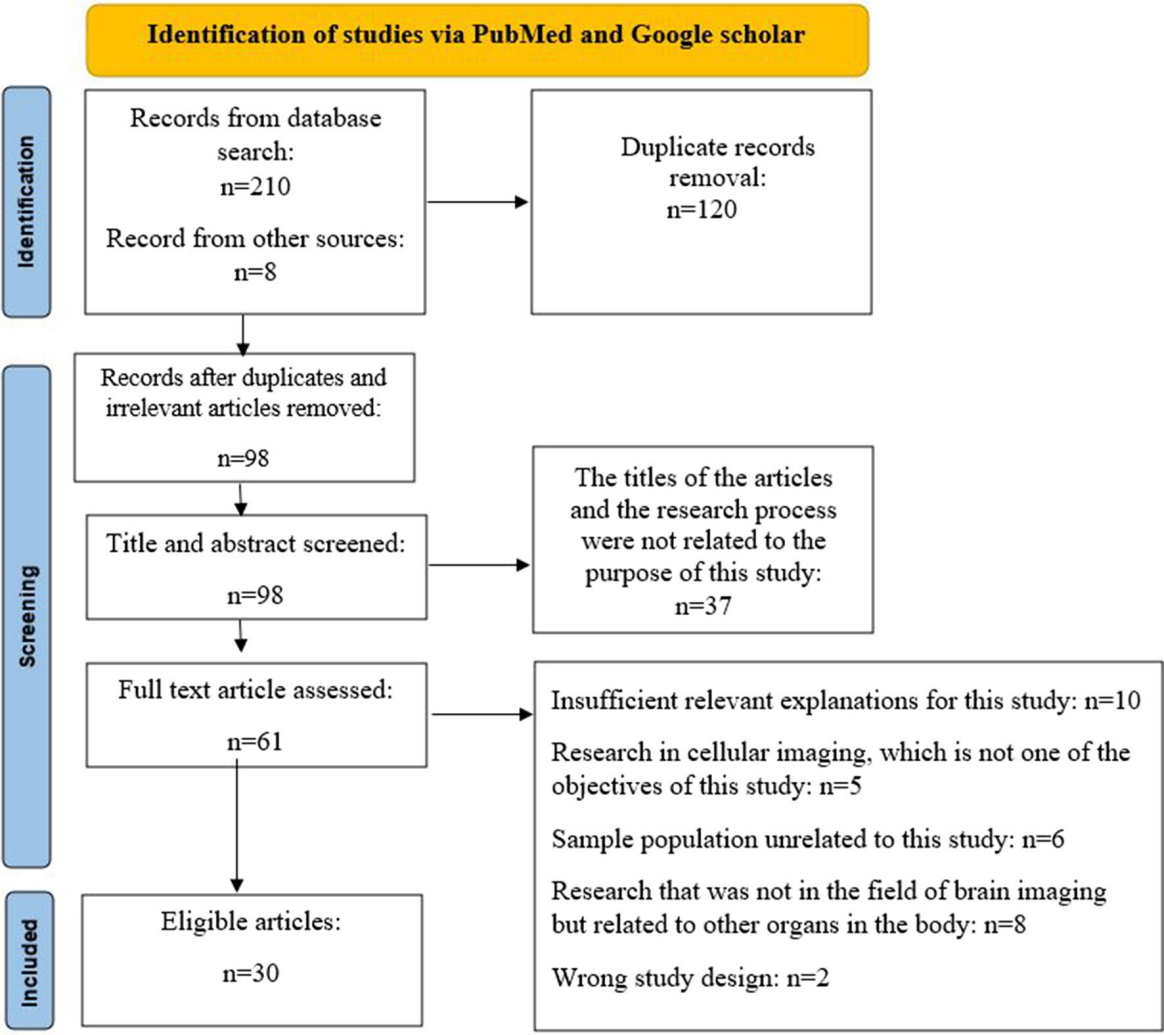
Follow-up search method based on PRISMA guideline

The Preferred Reporting Items for Systematic Reviews and Meta-Analyses (PRISMA) guidelines were the headline for selecting suitable articles [10] (Fig. 1).

Databases of PubMed and Google Scholar were searched to obtain the relevant articles for this paper.

The keywords used included Quantitative susceptibility mapping, amyloid-Beta PET, Tau PET, Alzheimer’s disease, Iron, Magnetic susceptibility, and basal ganglia (BG) from 2013 to 2021.

From the final cases, articles were selected that provided more relevant and complete explanations about the relationship between iron and neurodegenerative diseases and its imaging methods.

## Results

### Background

#### Iron

Iron is the most abundant non-diamagnetic element in the human brain, mainly stored as hemosiderin-6 or ferritin in the brain.

It can be transmitted between different brain parts along sections of nerve cells by non-transferrin-bound and transferrin-bound iron forms [11, 12].

Iron plays a crucial role in many biological functions of the body, such as cell growth and differentiation, protein expression, cyclin accumulation, and the production of reactive oxygen species [1].

Iron acts as a Co-factor for various enzymes involved in functions, such as oxygen transfer, electron transfer, neurotransmitter, and myelin production [13, 14].

**Fig. 2:**
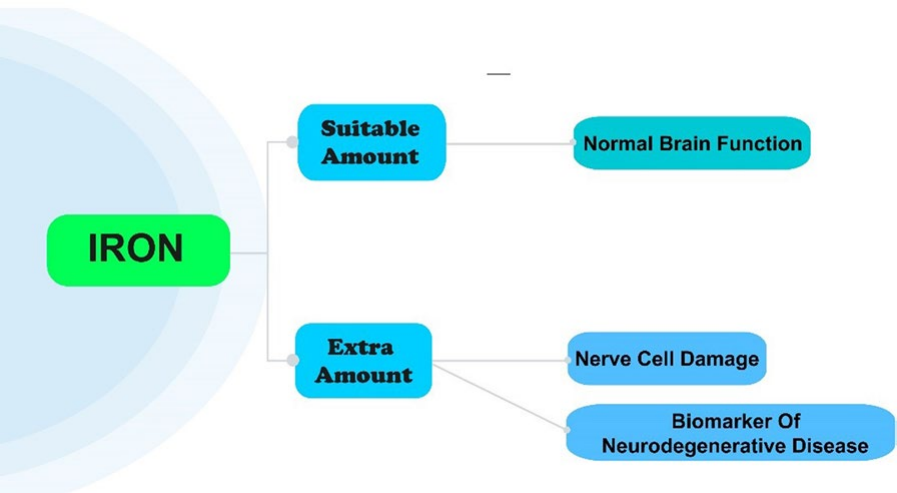
Consequences of the presence of iron with inappropriate amounts in the brain

Despite all the benefits of having iron in the body, excessive iron levels in the brain are seen in different roles in destroying the nervous system, leading to the production of toxic free radicals, resulting in oxidative damage (Fig. 2).

Toxic free radicals lead to membrane lipid peroxidation and lipofuscin accumulation in nerve cells [1].

Oxidative stress is associated with reducing the function of nerve cells [15] and selective cognitive decline in patients with or without dementia [16].

The presence of iron as one of the main components of senile plaques and neurofibrillary tangles has been proven [17].

In other words, iron concentration is an ideal condition for amyloid-beta (Aβ) aggregation and neurotoxicity [18].

According to a recent study by Ayton et al. [19], there is an association between iron accumulation in the inferior temporal gyrus (ITG) and the slope of cognitive decline in individuals with significant Aβ plaques and neurofibrillary tau tangles.

Excess iron leads to inflammation, which causes a decrease in myelin in the brain [20].

Previous studies have shown that excess iron deposition is closely associated with various neurodegenerative diseases like Alzheimer’s and Parkinson’s [21].

Iron gradually accumulates in structures such as the BG, hippocampus, cerebellar nucleus, and subcortical areas of the brain [22]; Still, its highest concentration is in deep gray matter (DGM) [23].

Measuring iron deposition in the brain can help explain the pathophysiological process of the disease, be used as a feature in the diagnosis of neurodegenerative disease [24], and basis for targeted treatment [25].

We are not yet sure whether iron deposition is the result or cause of neurodegenerative diseases. Still, today we know that monitoring the spatial distribution and temporal dynamics of iron deposition leads to a better understanding of the pathogenesis of the disease [26].

Therefore, quantification of iron deposits is critical; measuring the iron level in different brain parts over specific periods allows us to examine the sequences of disease events.

#### Myelin

According to the findings, iron is necessary for myelin production, and iron deficiency leads to hypomyelination [11, 20].

Recent studies have reported a negative relationship between iron levels and myelin content in the ventral striatum [27]. However, this relationship needs to be re-examined in the thalamus, BG, and white matter (WM) areas [28].

Myelin degeneration happens in some neurological diseases, such as Alzheimer’s disease (AD) and Parkinson’s disease (PD), which disrupts iron homeostasis in the brain [29].

However, understanding the relationship between iron concentration and myelin levels in different brain areas, like BG and internal capsule (IC)**, **is necessary to investigate the physiological mechanisms of normal aging and some neurodegenerative diseases.

#### Amyloid-beta plaque

As mentioned earlier, Aβ deposits are a significant feature of AD.

Proteins generally have high concentrations of paired electrons and are classified diamagnetic materials.

So, the accumulation of Aβ leads to an increase in electron density, changes in local susceptibility, and produces contrast to surrounding natural tissues.

On the other hand, brain iron is a group of paramagnetic substances.

Therefore, Aβ plaques and iron present in the substance have opposite effects on the magnetic susceptibility of the tissue [9].

#### Magnetic susceptibility

Magnetic susceptibility is an intrinsic and physical property of tissue representing the response of the body's magnetic material to an applied external magnetic field that reflects the composition of the tissue and is used to chemically identify and quantify substances such as iron and calcium and contrast agents [30].

Magnetic susceptibility of the tissue mainly depends on the properties of its content. It is greatly affected by substances such as iron, lipids, calcium, or myelin in the tissue.

Any change in the concentration and accumulation of these tissue compounds will have consequences.

Iron is the most crucial cause of Magnetic susceptibility changes in subcortical gray matter structures, so it is vital to measure the magnetic susceptibility of these areas of the brain as an indicator of iron deposition in neurodegenerative diseases [31].

Most biological substances, such as calcium or white matter myelin, cause negative susceptibility changes and are diamagnetic.

But iron stored in ferritin, hemosiderin, and neuromelanin in brain tissue, and iron embedded in deoxyhemoglobin in venous blood cause positive susceptibility changes and a strong magnetic field and are classified in the group of paramagnetic materials [32].

Based on the results of previous studies, the amount of iron in the DGM nuclei is well correlated with the magnetic susceptibility of the tissue [33].

Assessing changes in the magnetic susceptibility of brain tissue that occur for various reasons, such as the demyelination process, can give us a good insight into the pathological course of neurodegenerative diseases [34, 35].

The importance of using non-invasive techniques for this assessment is very significant.

### Methods for evaluating substances deposited in the brain and their advantages/disadvantages

Over time, different methods for this evaluation have been introduced, each of which has advantages and disadvantages.

There are some imaging techniques; Proton density, T1-weighted, T2-weighted, and T2*-weighted imaging are the routine sequences considered to quantify magnetic susceptibility and indicate the pathological course of the disease [36].

**Fig. 3:**
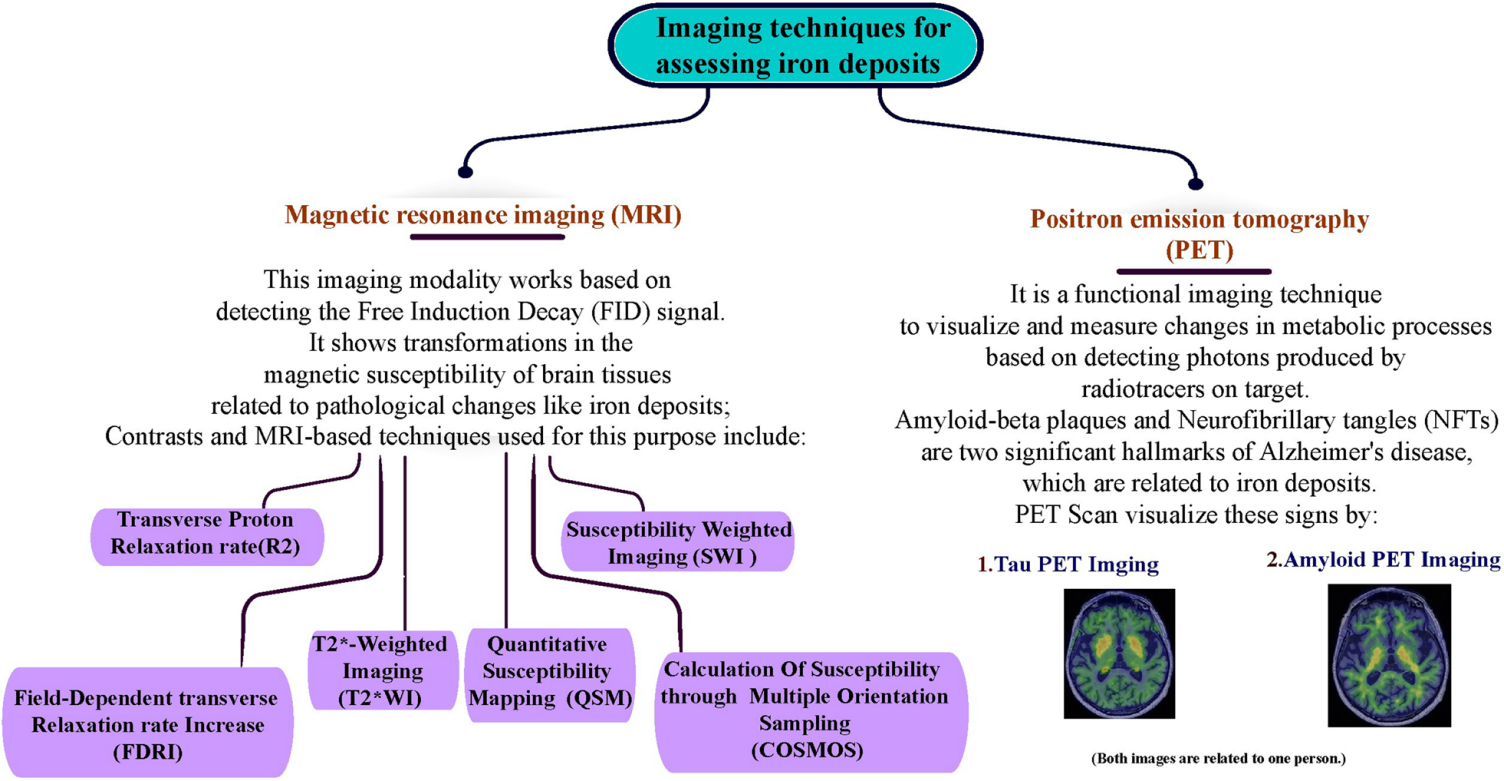
Methods for evaluating substances deposited in the brain

**Fig. 4:**
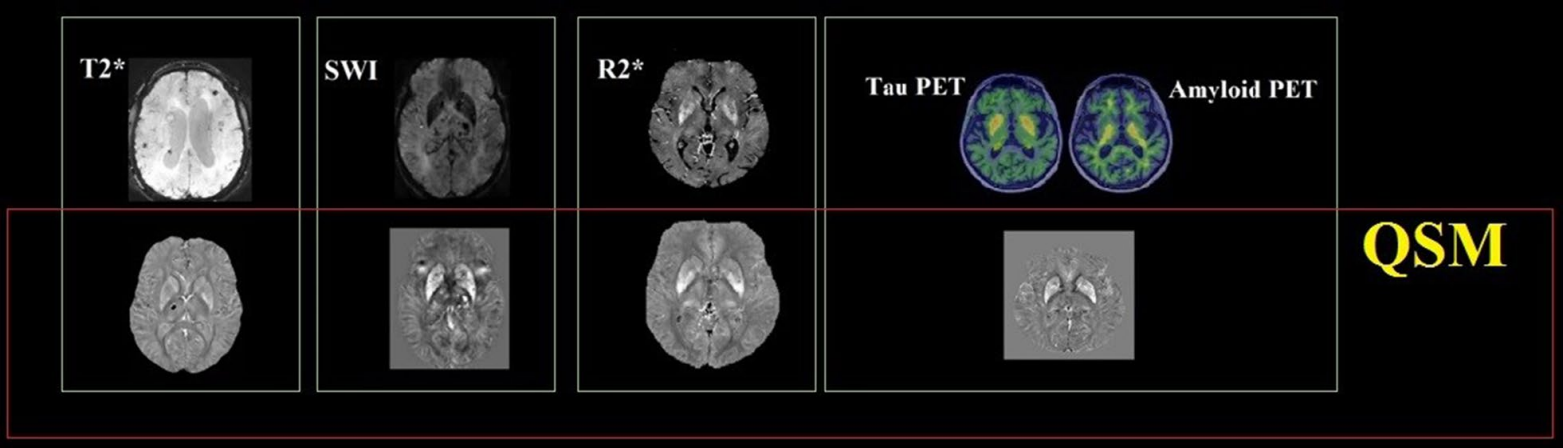
Presentation of images obtained from some imaging techniques to assess pathological changes in the brain

Todays, paramagnetic-deoxyhemoglobin-based fMRI [37], susceptibility weighted imaging [23], phase imaging [16], quantitative susceptibility mapping, and calculation of susceptibility through multiple orientation sampling (COSMOS) technique are used (Figs. 3, 4).

In addition to the methods mentioned, indirect detection of Aβ plaques is possible using MRI methods by injecting a contrast agent such as gadolinium (Gd) or monocrystalline-iron-oxide nanoparticles [38].

#### T2*-weighted imaging

T2*WI is a new technique based on multi-echo gradient recalled echo (GRE) sequences principles that acquires a gradient echo signal.

This contrast shows changes in tissue susceptibility and thereby creates an assessment of transformations in tissue content.

However, this imaging contrast depends on scanning parameters and object orientation.

Also, its magnitude and phase images contain blooming artifacts [39].

#### Susceptibility weighted imaging

SWI was introduced by Haacke et al. in 1991.

It is a post-processing strategy that produces susceptibility images obtained only from a single GRE pulse-echo sequence [40] and is the precursor of QSM [41].

This method has many clinical applications, such as cerebral microbleeds examination, hemorrhage, tumor detection, thrombosis, and stroke [42].

Nevertheless, this technique is weak in terms of quantitative validity because the resulting phase is mainly non-local and orientation-dependent. The amount of magnetic susceptibility of the surrounding tissue, the position of the individual's head, and high-pass filtration are some of the factors involved in creating these disadvantages [43, 44].

In addition, SWI images have the blooming artifact, and its quantification is not accurate [45].

#### Transverse proton relaxation rate

Transverse proton relaxation rate is a non-invasive method used to detect Aβ plaques and is linearly proportional to the amount of iron content [46].

This method can measure the interaction of T2*, which leads to the parameter R2*.

R2 ∗ method suffers from blooming artifacts and cannot measure tissue susceptibility correctly; in addition, results depend on tissue water content, tissue iron content, and scanner field strength [39].

#### Field-dependent transverse relaxation rate increase

FDRI is one of the first methods for assessing magnetic susceptibility changes of tissue and examining iron deposition in tissues [47].

In this method, R2 imaging performs with two different magnetic field strengths; the increase in relaxation in the higher field is attributed to iron and is a new way to measure iron stores in tissue [48].

This method has limited clinical application because it requires scanning the specified volume with two different magnetic field strengths [49]*.*

#### Calculation of susceptibility through multiple orientation sampling

Today, a strategy based on a single field strength called the calculation of susceptibility through multiple orientation sampling (COSMOS) has been introduced [50].

COSMOS magnetic susceptibility reconstruction is reliable, but the head needs to be scanned in different orientations [50].

#### Positron emission tomography

Today, positron emission tomography (PET) scans and radio tracers such as Fluorine-18 based compounds(18F) and carbon-*11*-labeled *Pittsburgh compound B* (*11C*-PIB) are used for in vivo amyloid-beta imaging at preclinical stages [51].

However, drawbacks of PET, including low resolution, high ionizing radiation, and high cost, make it reluctant to use for early screening and early detection of disease [52].

#### Quantitative susceptibility mapping

QSM is a newfound non-invasive developed MRI technique that can measure the magnetic susceptibility of human tissues [36].

It has the highest specificity and sensitivity for detecting and quantifying iron levels accumulated in the brain compared to other methods; it is possible to study the local tissue magnetic susceptibility property quantitatively [48].

QSM is preferable to the R2^∗^ or FDRI methods for evaluating the amount of iron deposition in the brain.

This technique fixes the blooming artifacts, does not depend on echo-time, water content, or field strength, and does not have many limitations mentioned in the previous methods [53, 54].

3D multi-echo gradient echo (mGRE) imaging method is a good sequence for QSM reconstruction [55].

Both phase and magnitude images of the mGRE sequence are used in this technique [48].

Different algorithms of QSM reconstruction steps use phase images to evaluate magnetic susceptibility differences between tissues, detect local variations in iron content, minimize magnetic field orientation dependence, and eliminate non-local effects compared to phase imaging [56].

**Fig. 5:**
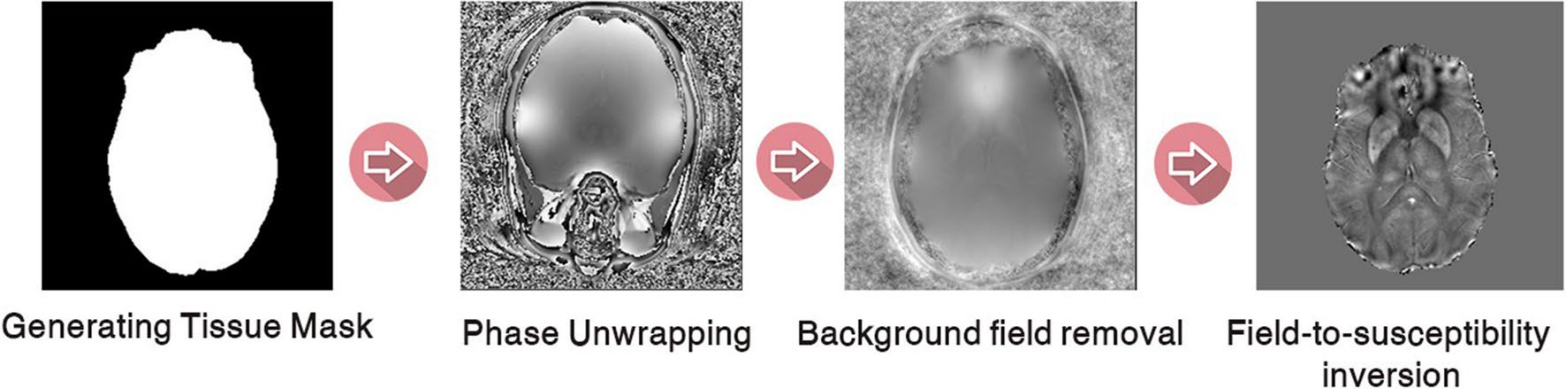
The four main steps of QSM reconstruction include generating tissue mask, phase unwrapping, background field removal, and field-to-susceptibility inversion

The four main steps of QSM reconstruction include generating tissue mask, phase unwrapping, background field removal, and field-to-susceptibility inversion; each of which can be performed by different algorithms (Fig. 5).

##### Quantitative susceptibility mapping disadvantages

The execution time of the multi-echo sequences is relatively long; it typically takes 5–10 min for the entire brain scanning, so it is a slow imaging technique.

Keeping the patient's head steady during this time is not an easy task, especially if the patient has a disease such as PD.

Therefore, depending on the patient physical and mental condition, this time is not appropriate for clinical exams [57].

### Clinical uses of QSM

The QSM technique to identify pathological changes in the brain has a wide range of applications.

The changes that lead to variances in the magnetic susceptibility of the tissue can be assessed using this technique.

**Fig. 6:**
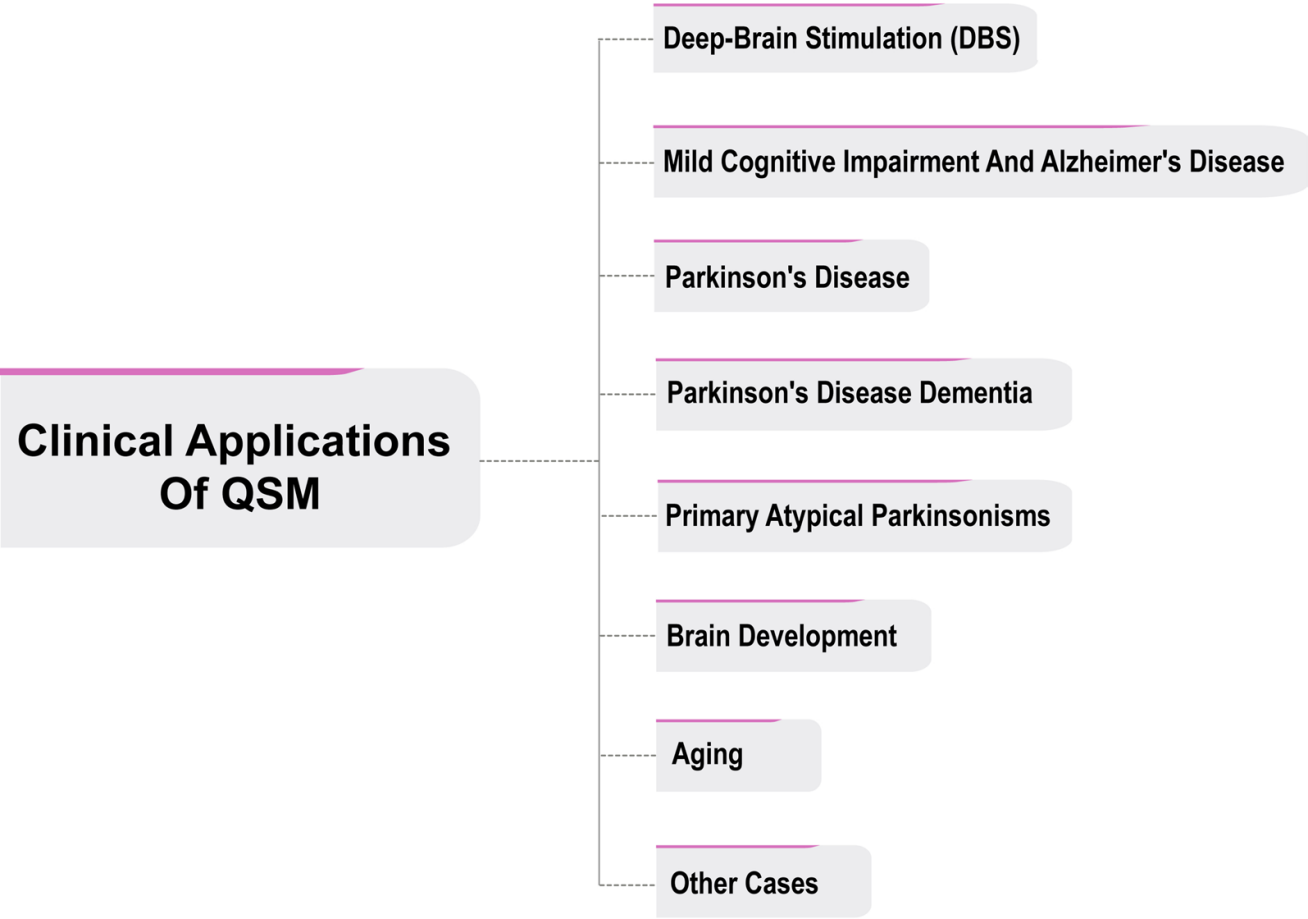
Clinical applications of QSM

For example, identifying the first brain changes in diseases such as Alzheimer's or Parkinson's, evaluating the process of brain changes during aging, or using this technique to place the relevant electrodes in deep-brain stimulation (DBS) surgery in the correct position, and so on (Fig. 6).

Also, in this section, the routine imaging techniques used in identifying these cases are discussed first. Then the advantages or disadvantages of the QSM technique in the same area are evaluated.

Finally, the proposed differentiation method between the studied groups based on the obtained QSM values ​​is introduced.

After reviewing recent studies, we have listed some critical parameters related to the execution of the MRI imaging sequence required to reconstruct the QSM technique in Table 1.

**Table 1:** Demographic information and MRI scans parameters related to research examined research in this study

Study	Sample size	Strength of MRI system	Sequence used	TR	TE
Au et al. [125]	13 Patients with early stage AD, 10 patients with late stage AD, and 30 healthy subjects	3 T	3D fast-field echo	45 ms	8echoes/*Δ*TE:5.2 ms/TE1:4.0 ms
Syam et al. [136]	26 Patients with PD, 27 patients with PSP, and 26 healthy subjects	3 T	3D multi-echo gradient-echo	62.2 ms	5 echoes/range 5.7–29.5 ms
Li et al. [132]	114 Healthy subjects	3 T	Gradient echo imaging	53 ms	40 ms
	336 Healthy subjects	3 T		25 ms	17.5 ms
	173 Healthy subjects	1.5 T		25 ms	17.5 ms
Cogswell et al. [126]	296 Healthy subjects, 69 patients with MCI, and 56 patients with amnestic dementia	3 T	3D-MEGRE	28 ms	6.7, 10.6, 14.5,18.4, and 22.4 ms
Fedeli et al. [127]	26 Patients with primary atypical parkinsonisms, and 49 patients with PD	3 T	3D spoiled multi-echo GRE sequences	36 ms	5, 12, 19, 26, and 33 ms
Shahmaei et al. [124]	30 Patients with PD and 15 healthy subjects	3 T	GRE T2*	38 ms	4 and 41.8 ms
Li et al. [122]	22 Patients with AD, 22 Patients with MCI, 25 Patients with SCD, and 25 healthy subjects	3 T	3D multi-echo gradient-echo	41.8 ms	16 echoes/*Δ*TE: 2.3 ms/TE_1_: 3.3 ms
Pu et al. [134]	16 Healthy adult macaques	3 T	3D multi-echo gradient-echo	60 ms	32 echoes/ΔTE = 1.42 ms/TE_1_: 2.4 ms
Spotorno et al. [135]	236 Amyloid-*b*-positive subjects, 78 cognitively unimpaired, and 158 cognitively impaired patients	3 T	3D multi-echo gradient-echo	24 ms	5.00, 8.80, 12.60, 16.40, and 20.20 ms
Spincemaille [139]	10 Healthy subjects	3 T	3D multi-echo gradient-echo	24.48 ms	5 echoes: 3.85, 7.97, 12.09, 16.21, and 20.33 ms
				45.08 ms	10 echoes: 3.85, 7.97, 12.09, 16.21, 20.33, 24.45, 28.57, 32.69, 36.81, and 40.93 ms
		7 T	3D multi-echo gradient-echo	24.55 ms	5 echoes: 3.81, 7.91, 12.00, 16.10, and 20.20 ms
				45.03 ms	10 echoes: 3.81, 7.91, 12.00, 16.10, 20.20, 24.29, 28.39, 32.48, 36.58, and 40.68 ms
Li et al. [131]	10 Healthy subjects	3 T	3D multi-echo gradient-echo	40 ms	6 echoes/ΔTE: 6 ms/TE_1_: 6 ms
Gong et al. [92]	4 Pairs of transgenic mice with abnormal beta amyloid-aggregation (Tg-SwDI)	7 T	3D multi-echo gradient-echo	250 ms	TE1: 3.72 ms /ΔTE: 5.52 ms/TE10: 53.36 ms
Du et al. [2]	30 Patients with AD	3 T	3D gradient-echo (GRE)	22.9 ms	3.2 ms
Li [129]	31 Non-demented PD patients, 10 patients with PDD and 27 healthy subjects	3 T	SWI with velocity-compensated 3D fast-field echo	TR/TE: 28/23 ms	
Kim et al. [121]	19 Patients with aMCI, 19 patients with mild and probable AD, and 19 healthy subjects,	3 T	3D fast field*-*echo (FFE)	43 ms	TE_1_: 3.4 ms/*Δ*TE: 6.0 ms/ TE_7:_ 39 ms
Wei et al. [140]	7 Healthy subjects	3 T	Standard flow-compensated 3D fast spoiled-gradient-recalled (SPGR)	TR/TE: 50/40 ms	
Ide et al. [128]	19 Patients with PD and 41 healthy subjects	3 T	3D multi-echo spoiled gradient echo (GRE)	58.4 ms	11 echoes/ΔTE: 5 ms*/*TE_1_: 4.5 ms
Moon et al. [133]	12 Patients with VaD, 27 patients with AD, and 18 healthy subjects	3 T	Susceptibility-weighted angiography sequence [SWAN]	37 ms	8echoes/ΔTE: 4.09 ms/TE_1_:3.5 ms
Sun et al. [57]	6 Healthy subjects	1.5 T	Standard gradient recalled echo (GRE) 3D-radiofrequency spoiled GRE	TR/TE: 49/40 ms	
Acosta-Cabronero et al. [21]	8 Patients with early-stage probable AD	3 T	Susceptibility-weighted-imaging	35 ms	20 ms

#### Deep-brain stimulation

DBS is a reversible stereotactic neurosurgery technique used for PD and other treatment-resistant neurological and psychiatric disorders [58].

Medial globus pallidus (GPm), centromedian nucleus (CM), and the centromedian-parafascicular nucleus complex are the most important goals for DBS [59, 60].

Globus pallidus (GP) separates into the medial globus pallidus (GPm) and the lateral globus pallidus (GPl) by a thin layer called medial medullary lamina (MML) [60, 61].

During the DBS process, electrodes must be located in specific target structures in the brain, then implanted brain pacemaker performs electrical stimulation [62, 63].

The effectiveness of DBS is related to the ability to accurately target determination because this target is different for each person anatomically [64].

MRI sequences such as T2-weighted fast-spin-echo [65] or proton density-weighted [66] are used to determine the target in DBS and static imaging and obtaining stereotactic imaging data [67].

Some factors cause difficulty to determine the CM coordinates from T1W or T2W images, such as low CM volume [59], lack of sufficient contrast between the CM and the surrounding tissues, so these sequences cannot visualize MML conclusively.

Todays, a normalized atlas is registered to the patient's MRI data to determine the CM coordinates for target localization [68].

The method of indirect targeting uses a normalized atlas which includes limitations because there are differences between the brain structures of different patients, which may lead to registration errors and increase surgical complications [69].

Due to these limitations, the lack of an imaging technique for accurate and direct visualization of CM and GPm makes it hard to target them for DBS surgery.

If the desired anatomical locations are visible with appropriate and specific contrast [70], we can improve the accuracy of the work by direct targeting.

Therefore, it is necessary to have a suitable imaging method to determine and image goals structures [71].

##### Routine imaging methods used to determine the target structure in DBS

High-resolution T1W images with about 14 h of scan time are used to show these substructures of the thalamus, but this method is not suitable for clinical use due to the long scan time [72].

Kanowski et al. showed that using two-dimensional high-resolution proton density-weighted images at 3 Tesla MRI scanners, CM can be identified in 13–26 min. Still, only a few slices covered the target area [73].

Bender et al. showed that by optimized 3D MPRAGE protocol, Relevant doctors could identify CM in 20 min, but thalamic substructures are not well distinguishable [74].

##### The role of quantitative susceptibility mapping in determining the target structure in this surgery

QSM is a reconstructed map of MRI phase images of a three-dimensional multi-echo gradient-echo sequence (3D mGRE).

This magnetic susceptibility map efficiently characterizes brain structures such as the CM region and GPm.

This technique uses magnetic field variations to calculate quantitative maps of magnetic susceptibility changes [71].

DBS goal structures such as the subthalamic nucleus and internal Globus pallidus are rich in iron (paramagnetic), and there is white matter (diamagnetic) around them [75–77].

QSM uses the susceptibility differences between contents and has been introduced as a suitable imaging method for CM detection.

Compared to R2* mapping, T2WI, T2*WI, phase imaging, and SWI, QSM images have superior contrast in the visualization of the GPm [78].

#### Mild cognitive impairment and Alzheimer's disease

Mild cognitive impairment (MCI) is a temporary high-risk stage before the onset of most neurodegenerative diseases [79].

Diagnosis at the MCI stage helps prevent the onset and progression of Alzheimer's disease [80].

AD is part of a large group of neurodegenerative diseases that seriously affect the quality of life of the elderly and impose a heavy economic burden on society.

In recent years, the incidence of AD has been increasing; the two most important clinical features of this disease are cognitive decline [81] and memory loss [82].

Early pathological changes occur in the early stages of the disease. As the disease progresses, symptoms such as learning disabilities and memory impairment develop, and we use clinical criteria to diagnose AD [52].

Today we have concluded that microscopic changes occur long before morphological changes and the onset of clinical signs [83]; introducing a reliable and sensitive biomarker at the microscopic and pathological level is required for early diagnosis and monitoring of disease progression [84].

Due to the lack of a reliable biomarker sensitive to these changes, the probable diagnosis of the disease occurs only in the advanced stages.

In the advanced stages of the disease, we also see morphological changes such as hippocampal atrophy, which can be identified using various MRI techniques [85].

Histochemical, histopathological, and imaging studies proved altered iron metabolism is associated with AD [86].

Senile plaques and tau neurofibrillary tangles are essential factors for developing AD. Aβ40 and Aβ42 are Aβ peptides that are most components of senile plaques commonly present in AD [87].

Today, the relationship between iron accumulation in AD and its association with Aβ aggregates and neurofibrillary tangles has been proven [86].

Based on evidence and studies, high iron levels lead to the overproduction of these Aβ peptides [88]; these peptides accumulate rapidly and form toxic oligomers and fibrils [89].

Consequently, plaque accumulation with accelerated oxidative stress leads to the loss of nerve cells [83].

Iron deposition and demyelination of WM increase magnetic susceptibility in a specific area of the brain [90].

##### Routine imaging methods used to diagnose Alzheimer's disease

Initial identification of brain plaques in Alzheimer's disease is possible with amyloid-beta and tau PET imaging.

Studies on the association between the QSM and the amyloid-beta PET results have shown that hyper-susceptibility occurs with age in areas of the cortex and deep gray nuclei [91].

Research projects have also shown differences in QSM values between people with AD and people with cognitive impairment or other conditions that damage the nervous system [25, 92].

Studies based on the simultaneous use of QSM and amyloid-beta PET to determine the association between iron deposition and Aβ accumulation have also been performed in ex-vivo brain samples and in-vivo mouse models and; the results have been acceptable [25, 34, 92].

##### The role of quantitative susceptibility mapping in the diagnosis of Alzheimer's disease

A wide range of neurodegenerative diseases related to demyelination, inflammation, microbleeds, and high iron deposition in the brain can be evaluated using the QSM technique [54].

Recent studies have also linked QSM-measured iron accumulation to cognitive decline and Aβ accumulation [93].

In addition, good linear correlations were observed in the BG iron content and QSM through post-mortem validation by mass spectrometry [94], X-ray emission and fluorescence[33], and Perls iron staining [95].

#### Parkinson's disease dementia

After AD, Parkinson's disease is the most common progressive neurodegenerative disease [96].

According to previous studies, the BG regulate the onset of motor activity.

A deficiency in the natural inhibition of the BG leads to the symptoms observed in PD [97].

*Dopamine* is a neurotransmitter [98] and acts in pathways such as the dopaminergic pathway from the substantia nigra (SN) to the caudate (Cd) and putamen (Pt) nuclei.

Abnormal deposition of iron in the deep gray nuclei of PD patients is the cause of dopaminergic neuron cell degeneration in the SN; after that, the production of dopamine stops, and the body's movements become irregular [99].

In the nervous system, α-synuclein is a presynaptic neuronal protein in various brain parts, such as the hippocampus, SN, neocortex, cerebellum, and thalamus.

Its abnormal accumulation in patients with PD leads to the formation of Lewy bodies.

Due to this feature, PD can be considered as a type of synucleinopathies [54].

On the other hand, according to a previous study by Langkammer et al. strong linear relationship was observed between the concentration of iron and the magnetic susceptibility value in the structure of gray matter (GM) [23].

One of the most common early non-motor manifestations of PD is dementia; as the disease progresses, symptoms associated with neurological disorders develop [96].

Dementia in PD may be associated with atrophy because MRI studies have shown structural changes in the amygdala and hippocampus in parkinson's disease dementia (PDD) patients [100].

PDD patients experience significant cognitive decline, including impaired executive function, visual function, attention, structural function, and memory.

Compared to AD, lesser deficits in language functions and more significant deficits in executive functions are observed in PDD [101].

According to the evidence, there is a link between limbic anomalies and symptoms of dementia.

##### Routine imaging sequences used to diagnose Parkinson's disease dementia

Researchers can use various MRI sequences to evaluate the mineralization of DGM and differentiate various neurodegenerative Parkinsonian disorders.

These sequences include R2, R2*, SWI, T2WI, and T2 * WI [93].

In this case, R2* imaging is more sensitive than T2 and T2 * weighted imaging [102].

The quality of the R2* imaging technique depends on the strength of the magnetic field; It has no authentic connection with iron accumulations.

Also, blooming artifact in this method increases with increasing time of echo (TE) [103].

#### Primary atypical parkinsonisms

Parkinsonism is a nervous system syndrome and has different symptoms such as tremors and instability, slowness of activity, and rigidity of muscles.

Primary *Atypical Parkinsonisms (*APPs) are conditions that have different categories such as Progressive Supranuclear Palsy (PSP), Corticobasal degeneration (CBD), and Multiple System Atrophy(MSA) [104].

PSP is the most typical fatal atypical Parkinsonian disorder with a median survival of around seven years [105].

There is currently no specific treatment for PSP other than symptomatic and supportive therapies, and tau-focused methods may be used in the future [106].

PSP has clinical features similar to diseases such as PD, Frontotemporal Dementia (FTD), and CBD [105, 106].

Today differentiating between PD and APPs is the issue of a challenge due to the similarity of symptoms in the early stages of these diseases [107].

The diagnostic method currently used is made on clinical grounds, and the need for a valid biomarker is required for rapid diagnosis of the disease [104].

##### Routine imaging sequences used to diagnose primary atypical parkinsonisms

The SWI sequence is used to diagnose APPs**, **but it does not accurately distinguish between PD and APPs.

Dorsolateral nigral is known as Nigrosome-1, which is considered a sensitive marker of degenerative parkinsonism; The Nigrosome-1 hyperintensity is lost using the SWI sequence.

Therefore, a reliable biomarker in imaging is essential to differentiate between PD,PDD, APPs, and other movement disorders [108].

##### The role of quantitative susceptibility mapping in the diagnosis of Parkinson's disease dementia and primary atypical parkinsonisms

In Parkinson's disease spectrum, there are different patterns of magnetic susceptibility in deep gray nuclei.

APPs patients can be distinguished from PD patients due to different patterns of iron deposition.

QSM is a non-invasive technique that uses magnetic resonance imaging to detect changes in magnetic susceptibility and quantify the amount of iron deposition in the brain [109].

QSM has higher accuracy than other methods[110]. Strong correlations between QSM values and tissue iron content of DGM structure have been observed by post-mortem studies [23].

#### Brain development

According to Li et al., the WM becomes more diamagnetic as the brain develops, from 1 to 83 years old.

During the early phases of brain development, QSM can be used to monitor loss or delayed myelination.

Magnetic susceptibility anisotropy (MSA) is one of the QSM techniques that measure myelin concentration and progression of myelination in the postnatal brain.

A Rapid decrease in Magnetic susceptibility anisotropy values or no improvement represents different conditions such as dysmenorrhea or hypomyelination disorders [111].

#### Aging

During the aging process, a gradual increase in the magnetic susceptibility values occurs as a natural physiological process due to iron accumulation and myelin breakdown.

In a recent study on using QSM for evaluating age-related changes, a nonlinear increase of susceptibility with aging was perceived in the globus pallidus, red nucleus (RN), SN, and dentate nucleus (DN).

Also, a linear increase in magnetic susceptibility was observed in structures like Pt and Cd [112].

#### Other QSM clinical applications

In addition to the above, QSM has other clinical uses such as investigation of iron deposition and susceptibility changes in the WM in neurodegenerative diseases such as FTD, Vascular Dementia (VaD), Huntington's Disease (HD), Wilson's Disease, and motor neuron disease (MND) [77].

This technique is also used in neurovascular disorders fields such as traumatic brain injury (TBI), venous oxygen saturation, inflammatory/demyelination disease, assessing brain tumor due to its differentiation of calcification and hemorrhage[113], cerebellar Ataxia, imaging of traumatic intracranial hemorrhage, and monitoring multiple sclerosis patients without the need for Gd [114].

Other areas of use of this method are the evaluation of tissue function in diseases [115],MR venography, checking cerebral cavernous malformations [116], mapping rate of cerebral metabolic [117], mapping magnetic nanocarrier distribution [118], accurate measurement of liver iron concentration [119] and differentiation diamagnetic materials such as calcium from paramagnetic materials such as iron [120].

#### The methods of differentiation between the studied groups, based on QSM values

The research projects in this field have only compared the iron deposition in the brain nuclei between the studied groups; by reviewing the articles, an increase in iron deposition in several brain nuclei was observed during some cognitive disorders, which can act as biomarkers to differentiate these disorders (Table 2).

**Table 2:** Brain nuclei suffer from increased iron deposition in various cognitive disorders (bold = increase QSM values)

		Caudate nucleus		Putamen nucleus		Hippocampus nucleus		Thalamus nucleus	
		QSM values (ppm)							
Moon et al. [133]	AD (*n* = 27)	**0.08344 ± 0.02244** ***p* = 0.002**		**0.0989 ± 0.03363** ***p* < 0.001**				0.04176 ± 0.02119	
	VaD (*n* = 12)	0.0859 ± 0.01369		0.09481 ± 0.0297				0.03129 ± 0.0151	
	CN (*n* = 18)	0.06396 ± 0.01638		0.05848 ± 0.02401				0.03671 ± 0.01895	
Kim et al. [121]	AD (*n* = 19)			0.00377 ± 0.002851		**− 0.020649 ± 0.002052** ***p* = 0.0010**		**− 0.019915 ± 0.002081** ***p* = 0.0060**	
	aMCI (*n* = 19)			0.002047 ± 0.002789		− 0.02655 ± 0.002008		− 0.023543 ± 0.002036	
	CN (*n* = 19)			− 0.003238 ± 0.002047		− 0.032943 ± 0.002099		− 0.030153 ± 0.002129	
Du et al. [2]	AD (*n* = 30)	**(No quantitative information was provided)**		**(No quantitative information was provided)**					
	CN (*n* = 30)								
Li et al. [122]	AD (*n* = 22)	**0.046 ± 0.011** **P = 0.00**		**0.089 ± 0.024** ***p* = 0.00**		− 0.022 ± 0.006		0.005 ± 0.013	
	MCI (*n* = 22)	**0.044 ± 0.009** **P = 0.00**		**0.071 ± 0.013** ***p* = 0.00**		− 0.020 ± 0.008		0.005 ± 0.013	
	SCD (*n* = 25)	**0.037 ± 0.006** ***p* = 0.00**		**0.063 ± 0.012** ***p* = 0.00**		− 0.018 ± 0.009		− 0.002 ± 0.011	
	CN (*n* = 25)	0.023 ± 0.019		0.031 ± 0.024		− 0.023 ± 0.009		− 0.004 ± 0.011	
Julio Acosta-Cabronero et al. [21]	Early stage probable AD (*n* = 8)	**(No quantitative information was provided)**		**(No quantitative information was provided)**					
	CN (*n* = 11)								
Tiepolt et al. [91]	AD (*n* = 10)	R	L	R	L	R	L	R	L
				0.0047 ± 0.0031	0.0031 ± 0.0041	− 0.0028 ± 0.0045	− 0.0020 ± 0.0046	0.0024 ± 0.0036	0.0029 ± 0.0040
	CN (*n* = 10)			0.0613 ± 0.0092	0.0482 ± 0.0052	− 0.0775 ± 0.0070	− 0.0513 ± 0.0072	0.0243 ± 0.0039	0.0328 ± 0.0060
Meineke et al. [141]	Mild-AD (*n* = 29)	AD versus HC)difference(							
		**0.0392** ***p* < 0.05**	**0.0232** ***p* < 0.05**		0.8879		0.3689		
	MCI (*n* = 31)	MCI vs HC)difference(							
		0.6026	0.2065		0.7037		0.4509		
	CN (*n* = 41)								
Kan et al. [142]	AD (*n* = 38)	**(No quantitative information was provided)**				**(No quantitative information was provided)**			
	CN (*n* = 36)								
Shahmaei et al. [124]	PD (*n* = 30)	0.153 ± 0.027	0.152 ± 0.022				**0.119 ± 0.012** ***p* = 0.005**	**0.239 ± 0.021** ***p* < 0.001**	
	CN (n = 15)	0.155 ± 0.011	0.163 ± 0.032				0.108 ± 0.008	0.146 ± 0.026	
Syam et al. [136]	PD (*n* = 26)	0.042 ± 0.0097	0.0404 ± 0.0111					**0.1108 ± 0.0205** *p* = 0.007**	
	PSP (*n* = 27)	**0.0569 ± 0.0121** ***p* < 0.001**	**0.0641 ± 0.0186** ***p* < 0.001**					**0.1443 ± 0.0301** ***p* < 0.001**	
	CN (*n* = 26)	0.0408 ± 0.0083	0.0401 ± 0.0118					0.0852 ± 0.0149	
Li et al. [129]	PD (*n* = 31)					**(No quantitative information was provided)**	**(No quantitative information was provided)**		
	PDD (*n* = 10)					**(No quantitative information was provided)**			
	CN (*n* = 27)								

		Substantia nigra cleus		Amygdala nucleus		Globus pallidus nucleus	
		QSM values (ppm)					
Moon et al. [133]	AD (*n* = 27)					0.13863 ± 0.03624	
	VaD (*n* = 12)					0.13195 ± 0.02112	
	CN (*n* = 18)					0.12678 ± 0.03244	
Kim et al. [121]	AD (*n* = 19)			**− 0.019073 ± 0.002293** ***p* = 0.0010**		0.044097 ± 0.004568	
	aMCI (*n* = 19)			− 0.027567 ± 0.002243		0.038721 ± 0.004468	
	CN (*n* = 19)			− 0.032889 ± 0.002345		0.03804 ± 0.004672	
Du et al. [2]	AD (*n* = 30)						
	CN (*n* = 30)						
Li et al. [122]	AD (*n* = 22)					**0.084 ± 0.015** ***p* = 0.00**	
	MCI (*n* = 22)					**0.063 ± 0.015** ***p* = 0.00**	
	SCD (*n* = 25)					**0.053 ± 0.012** **P = 0.00**	
	CN (*n* = 25)					0.033 ± 0.024	
Julio Acosta-Cabronero et al. [21]	Early stage probable AD (*n* = 8)			**(No quantitative information was provided)**			
	CN (*n* = 11)						
Tiepolt et al. [91]	AD (*n* = 10)	R	L	R	L	**R**	**L**
				− 0.0097 ± 0.0073	− 0.0056 ± 0.0063	**− 0.0085 ± 0.0088** ***P* < 0.0001**	**− 0.0071 ± 0.0116** ***P* < 0.0001**
	CN (*n* = 10)			− 0.0942 ± 0.0089	− 0.0637 ± 0.0111	0.2765 ± 0.0181	0.2929 ± 0.0140
Meineke et al. [141]	Mild-AD (*n* = 29)						
						0.8163	
	MCI (*n* = 31)						
						0.1110	
	CN (*n* = 41)						
Kan et al. [142]	AD (*n* = 38)			**(No quantitative information was provided)**			
	CN (*n* = 36)						
Shahmaei et al. [124]	PD (*n* = 30)						**0.247 ± 0.028** ***p* < 0.001**
	CN (*n* = 15)						0.178 ± 0.027
Syam et al. [136]	PD (*n* = 26)						0.1075 ± 0.021
	PSP (*n* = 27)						**0.135 ± 0.0321** ***p* = 0.004**
	CN (*n* = 26)						0.1058 ± 0.0116
Li et al.[129]	PD (*n* = 31)						
	PDD (*n* = 10)						
	CN (*n* = 27)						

Nevertheless, no specific value has been introduced for differentiation between these groups based on the QSM values ​​of various brain regions and different articles have used different methods to achieve this goal.

In general, the Receiver operating characteristic (ROC) curve analyses can differentiate between healthy individuals and the study group in the QSM technique, which requires appropriate Cut-Off values.

So far, no standard ​​cut-off values ​​have been announced, and researchers can use statistical software such as MedCalc (www.medcalc.org, Ostend, Belgium) [121, 122], Excel, or other ways [123, 124] to achieve this goal.

There are different steps and different algorithms for QSM reconstruction that have been used in various research projects [2, 15, 21, 92, 121–136].

#### Post mortem confirmation study to assess the validity of QSM technique

In 2012, a study was performed to determine the accuracy of the QSM technique for measuring iron deposits in the brain using the QSM technique and inductively coupled plasma mass spectroscopy of post mortem tissue specimens [54].

Based on the estimated brain tissue density of 1.04 g/cm^3^ and the effective number of Bohr magnetons (3.78), the contribution of ferritin paramagnetic to tissue susceptibility was estimated to be approximately 0.00132 ppm * [Fe] at 36.5 °C [137].

Since most of the iron in the brain is bound to ferritin proteins, it can be concluded that magnetic susceptibility is sensitive to changes in the concentration of iron in the human brain [22].

However, some factors confuse the results and can have a detrimental effect on the magnetic sensitivity of the tissue, such as iron in oligodendrocytes, which play a significant role in myelination, the abundance of other trace elements, Substances such as deoxygenated blood, transferrin, hemosiderin, myelin, calcium [71] and orientation dependency of susceptibility [138].

## Conclusion

There is a wide range of conditions needed to examine magnetic susceptibility changes in the brain.

Different methods each have advantages and disadvantages, but the QSM technique performs relatively well compared to other methods.

This method can be applied to some routine sequences in an MRI scanner and is independent of the field's shape, echo time, direction, and strength.

It has higher spatial contrast than R2 * and higher sensitivity compared to FDRI.

Considering the review of all methods of measuring and evaluating iron deposition in different brain areas, selecting the QSM method for clinical application is a good choice.

However, more extensive studies are required on the clinical use of this technique.

## Data Availability

Since this is a review article, it is based on recent research. These researches are included in the references.

## Abbreviations

3D mGRE: Three-dimensional multi-echo gradient-echo sequence (3D mGRE)
Aβ: Amyloid-beta plaques
AD: Alzheimer's disease
APPs: Primary atypical parkinsonisms
BG: Basal ganglia
CBD: Corticobasal degeneration
Cd: Caudate nucleus
CM: CentroMedian nucleus
DBS: Deep-brain stimulation
DGM: Deep gray matter
DN: Dentate nucleus
FDRI: Field-dependent relaxation rate increase
FTD: Frontotemporal dementia
Gd: Gadolinium
GM: Gray matter
GPm: Medial globus pallidus
GPl: Lateral globus pallidus
HD: Huntington’s disease
ITG: Lower temporal gyrus
MCI: Mild cognitive impairment
mGRE: Multi-echo gradient echo
MML: Medial medullary lamina
MND: Motor neuron disease
MRI: Magnetic resonance imaging
MSA: Magnetic susceptibility anisotropy
MSA: Multiple system atrophy
PD: Parkinson's disease
PDD: Parkinson's disease dementia
PET: Positron emission tomography
PSP: Progressive supranuclear palsy
Pt: Putamen
QSM: Quantitative susceptibility mapping
R2*: Relaxation rate
RN: Red nucleus
SN: Substantia nigra
SWI: Susceptibility weighted imaging
T1W: T1-Weighted
T1WI: T1-weighted imaging
T2WI: T2-weighted imaging
T2*WI: T2*-weighted imaging
TE: Time of echo
T2*W: T2*-weighted
T2WI: T2-weighted
TBI: Traumatic brain injury
VaD: Vascular dementia
WM: White matter
